# Neural networks underlying implicit and explicit moral evaluations in psychopathy

**DOI:** 10.1038/tp.2015.117

**Published:** 2015-08-25

**Authors:** K J Yoder, C Harenski, K A Kiehl, J Decety

**Affiliations:** 1 Department of Psychology, University of Chicago, Chicago, IL, USA; 2 Department of Psychology, University of New Mexico, Albuquerque, NM, USA; 3 Department of Neuroscience, University of New Mexico, Albuquerque, NM, USA; 4 Mind Research Network, Albuquerque, NM, USA; 5 Department of Psychiatry and Behavioral Neuroscience, University of Chicago, Chicago, IL, USA

## Abstract

Psychopathy, characterized by symptoms of emotional detachment, reduced guilt and empathy and a callous disregard for the rights and welfare of others, is a strong risk factor for immoral behavior. Psychopathy is also marked by abnormal attention with downstream consequences on emotional processing. To examine the influence of task demands on moral evaluation in psychopathy, functional magnetic resonance imaging was used to measure neural response and functional connectivity in 88 incarcerated male subjects (28 with Psychopathy Checklist Revised (PCL-R) scores ⩾30) while they viewed dynamic visual stimuli depicting interpersonal harm and interpersonal assistance in two contexts, implicit and explicit. During the implicit task, high psychopathy was associated with reduced activity in the dorsolateral prefrontal cortex and caudate when viewing harmful compared with helpful social interactions. Functional connectivity seeded in the right amygdala and right temporoparietal junction revealed decreased coupling with the anterior cingulate cortex (ACC), anterior insula, striatum and ventromedial prefrontal cortex. In the explicit task, higher trait psychopathy predicted reduced signal change in ACC and amygdala, accompanied by decreased functional connectivity to temporal pole, insula and striatum, but increased connectivity with dorsal ACC. Psychopathy did not influence behavioral performance in either task, despite differences in neural activity and functional connectivity. These findings provide the first direct evidence that hemodynamic activity and neural coupling within the salience network are disrupted in psychopathy, and that the effects of psychopathy on moral evaluation are influenced by attentional demands.

## Introduction

Psychopathy is a personality disorder associated with a constellation of traits including a lack of guilt and empathy, narcissism, superficial charm, dishonesty, reckless risk-taking and impulsive antisocial behavior.^1, 2^ Dysfunctional emotional processing is also a characteristic feature of psychopathy and is accompanied by atypical anatomical and functional connectivity between the amygdala and ventromedial prefrontal cortex,^3, 4^ as well as anomalous neural activity in regions such as anterior cingulate cortex (ACC), anterior insula (aINS) and the amygdala in response to affective stimuli.^3, 5, 6, 7, 8, 9, 10^ Given the importance of emotion and affective arousal in moral reasoning^11, 12^ (although some debate this^13, 14^), and work showing that the interpersonal/affective characteristics of psychopathy facilitate immoral behavior,^1^ studying individuals with various levels of psychopathy constitutes an important test case for understanding the neural mechanisms underpinning moral cognition and decision-making.

For instance, the literature on morality has been dominated by moral dilemmas, with early neuroscience investigations arguing for separate cognitive and affective processes.^15^ Some have advocated for using such dilemmas in psychopathy,^16^ as psychopaths are often thought of as having an intact ability to make inferences about another person's mental states, and a cognitive understanding of what is morally right or wrong,^17, 18^ although this effect is not always replicated.^19^ Several studies have reported abnormally utilitarian moral judgments in individuals with high levels of psychopathy personality traits^20, 21^ or in neurological patients with damage of the ventromedial prefrontal cortex (vmPFC).^22, 23, 24^ Incarcerated individuals with higher Psychopathy Checklist Revised (PCL-R) scores are more likely to endorse utilitarian solutions^25^ (although several small studies failed to find this effect^26, 27^). Most of these results fit with previous theoretical and empirical work documenting a key role of emotion in moral reasoning, especially the processing of distress signals and associations with action outcomes,^28, 29^ and pointing out that cognitive processing alone is insufficient to guide moral judgment.^30^ Thus, if an individual does not possess a capacity for experiencing affiliative prosocial emotions to accompany or guide their actions and predict their consequences on others, having only an explicit knowledge of moral norms may be insufficient to motivate moral and caring behaviors.^31, 32^

Taken together, these studies are consistent with the notion that psychopathy is characterized by an over-reliance on cognitive deliberation because these individuals lack an intuitive affective aversion to harming others.^33, 34^ However, limiting investigations to hypothetical dilemmas cannot provide a full account of atypical moral reasoning in psychopathy,^35^ and, even in the absence of behavioral differences, this disorder is marked by abnormal neural recruitment and anatomical connectivity.^26, 36^ Furthermore, some of these dilemmas are problematic and tell us very little about moral decision-making in everyday life.^37^ Several neuroimaging studies have indeed demonstrated that criminal psychopaths show greater hemodynamic activity in the lateral prefrontal cortex during processing of emotional stimuli than do non-psychopaths, and this finding is usually interpreted as either representing compensatory processes for deficient paralimbic activity or top-down cognitive control of salient stimuli.^8, 38^ Moreover, a strong distinction between affect and cognition may be a false dichotomy, both at the psychological and neurobiological levels.^39^ For instance, although it may be possible to assign especially affective or cognitive roles to specific areas of the cortex or limbic system, many 'affective' and 'cognitive' regions such as the hypothalamus, vmPFC, ACC, amygdala and lateral prefrontal cortex have widespread overlapping structural and functional reciprocal connections.^40^

Taking into account the large-scale nature of neural connections and adopting a network view of brain function has been an important step to understand how the nervous system supports complicated mental and behavioral activities.^41^ In conjunction with studies of neurological patients and community samples, a complex picture is emerging, wherein moral cognition involves the interaction of automatic processes, which support intuitions that are usually affectively laden, and controlled processes, which support deliberation and reasoning.^15, 42^ Importantly, these complementary computational systems arise from partially overlapping neural networks that support domain-general processes such as affective arousal, perspective-taking, attention, decision-making and motivational salience.^10, 15, 23, 29, 43, 44^ Nodes of these networks, which are consistently implicated in functional magnetic resonance imaging studies of moral reasoning, include the amygdala, aINS, ACC and vmPFC, as well as the posterior superior temporal sulcus (pSTS/TPJ), dorsolateral prefrontal cortex (dlPFC) and posterior cingulate.^45^ The right temporoparietal junction (rTPJ), in particular, has received a great deal of focus, as this region is involved with not only theory of mind^46^ and moral judgment^47, 48, 49, 50^ but also with reorienting responses and biological motion perception.^51^ This region is therefore a primary candidate in processing morally laden information. Also of particular interest for this study is the salience network, which is anchored by the dorsal aspects of the ACC and aINS, and extends across many regions, including the superior temporal pole, supplementary motor area, amygdala, ventral striatopallidum, hypothalamus, dorsomedial thalamus, and periaqueductal gray and ventral tegmental area.^10^ This network facilitates attention allocation toward personally or motivationally salient information.^44^ In the context of moral judgments, this salience system is also responsible for dynamically orchestrating shifts between cognitive control and default mode networks.^52^

Thus, an important next step in clinical neuroscience is to investigate the extent to which psychopathic traits relate to typical and atypical recruitments of these networks in the context of moral evaluations. Previous neuroimaging studies that investigated the impact of psychopathic traits on the salience network have produced inconsistent findings. Some studies reported that individuals with psychopathy fail to attach the appropriate significance to the distress cues of others, and show decreased hemodynamic response in the aINS and amygdala.^53^ However, in other contexts psychopathy has been linked to greater activity in these regions.^6, 54^ One appealing resolution to this apparent contradiction proposes that selective attention is also abnormal in psychopathy.^55^ In non-moral contexts, such as fear-potentiated startle, both behavioral and amygdala activity differences between groups were found to be reduced by focusing attention to threat-relevant information.^56^ This fits with recent research indicating that individuals with high and low psychopathic traits differ in the way they modulate attention to morally or socially relevant information,^57^ depending on their current goals and mental states. On this view, both bottom-up automatic and top-down controlled processes are disrupted in psychopathy. However, no study to date has directly manipulated task goals with respect to morally relevant information in the same individuals with different levels of psychopathy.

The current study was designed to investigate how psychopathy, both as a clinical taxon and personality dimension, influences neural encoding of moral valence in contexts when moral content is task-relevant or task-irrelevant. As discussed above, a great deal of work has already been performed on the neural networks that support explicit moral reasoning. Implicit moral processing, on the other hand, has received less attention in the neuroimaging literature, although there have been a few investigations.^58, 59, 60, 61, 62^ Implicit moral judgments correspond to decisions focusing on non-relevant aspects of the stimuli (for example, gender, age and location) when perceiving morally laden stimuli.^59^ In healthy individuals, morally relevant information is especially salient and therefore influences processing at multiple stages, even when it is not task-relevant.^11^ Examining both explicit and implicit processes in psychopathy can help to distinguish between two of the primary competing hypotheses about socioemotional processing in psychopathy, namely, whether psychopathy is marked by a failure to spontaneously encode task-irrelevant moral information as salient, or a failure to appropriately respond to such information.

To our knowledge, only one neuroimaging study has directly contrasted explicit and implicit moral processing tasks in its design,^62^ and reported an augmentation in hemodynamic response in the vmPFC during the explicit condition, and greater dlPFC activity in the implicit condition. The present study assesses implicit and explicit processing of moral information by having the same participants evaluate the stimuli during two tasks. Two regions of particular interest are the amygdala and right pSTS/TPJ. Historically, the amygdala has been argued to signal and prioritize the affective relevance of stimuli, regardless of whether or not it is task-relevant,^63^ and to provide a route for this salient information to influence downstream processing in other cortical regions, largely independent of top-down attention control.^64^ However, recent investigations have shown that the amygdala is not completely immune from top-down influences,^56^ and this has relevance to atypical affective processing in psychopathy.^55, 65^ Moreover, because the amygdala is highly interconnected with much of the cortex, functional connectivity seeded in the amygdala is expected to reveal distinct patterns of connectivity in explicit and implicit moral contexts. Functional connectivity seeded in the rTPJ was also assessed because, in addition to its previously discussed role in multiple processes important for sociomoral cognition, several studies have found an association between psychopathy and rTPJ/STS gray matter abnormalities.^66, 67^

We hypothesized that during implicit moral evaluation, where the presence or absence of harm is not task-relevant, psychopathy will be inversely related to neural activity in nodes of the salience network. Similarly, we predicted that psychopathy would lead to reduced functional connectivity seeded in both right amygdala and right TPJ to widespread cortical and limbic areas, especially to core nodes of the salience network (that is, dACC, aINS).^68^ During the explicit moral evaluation task, because psychopaths lack an intuitive aversive response to harm, they are expected to rely more on cognitive (controlled) computations, as evidenced by increased recruitment at the whole-brain level of prefrontal regions, rather than regions that support rapid processing, such as parahippocampus, amygdala, ventral ACC and brainstem. Moreover, this cognitive processing style is expected to require greater reliance on the integrative capacities of ACC, as evidenced by increased functional connectivity to this region from the right TPJ and amygdala.

## Materials and methods

### Participants

Overall, 112 male volunteers, all of whom were incarcerated in medium-security North American correctional facilities, participated in the study. A total of 21 participants were excluded from analysis because of lifetime threshold for either bipolar disorder or major depressive disorder (*n*=6), transferred to another site before PCL-R scores could be collected (*n*=8), excessive movement in the magnetic resonance imaging (*n*=7), poor task performance (*n*=2) or failure to complete the task (*n*=1). Thus, the final sample consisted of 88 male subjects (31.2±7.3 years). The study was approved by the Institutional Review Boards from the University of Chicago and the University of New Mexico.

Trained research assistants conducted PCL-R assessments, including file review and interview. Intelligence quotient (IQ) was assessed via Wechsler Adult Intelligence Scale. Power analyses indicated that for a medium effect size of 0.5, groups of at least 27 were required to achieve power greater than 0.8.^69^ Collection was continued until groups of this size were obtained. Participants with PCL-R scores of 30 or above were assigned to the high-psychopathy group (*n*=28, 32.6±7.4 years, IQ=94.2±12.9), whereas participants with scores of less than 20 were assigned to the low-psychopathy group (*n*=32, 30.1±7.2 years, IQ=96.8±12.6). Groups did not differ in terms of age, IQ or accuracy on either the implicit or explicit tasks (all *P*>0.2). Participants provided informed written consent and were compensated with pay consistent with the facility hourly labor wage. Inclusion criteria were IQ greater than 70 and age less than 50 years.

### Stimuli and task

**Figure 1:**
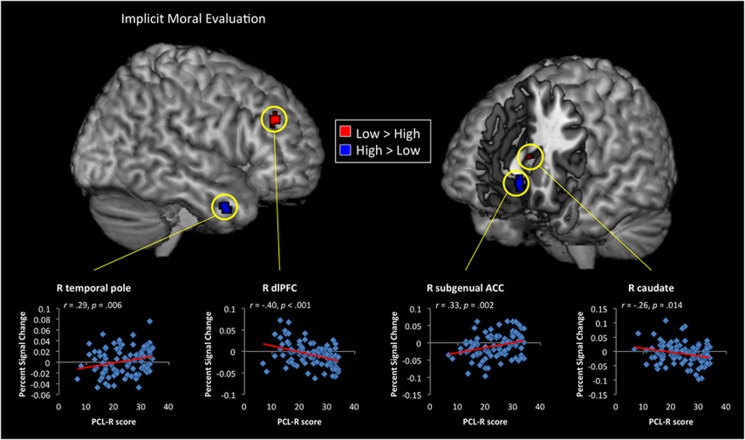
Example stimuli and whole-brain results for the Implicit Task. Examples of the final picture of helpful and harmful scenarios. Whole-brain contrasts showing significant (*P*<0.005) differences between High (Psychopathy Checklist Revised (PCL-R)⩾30) and Low (PCL-R⩽20) psychopathy groups during the Implicit Task. Below, scatterplots correlating PCL-R score against percent signal change within specific regions across all participants. ACC, anterior cingulate cortex; dlPFC, dorsolateral prefrontal cortex; R, right.

In the scanner, participants viewed scenes depicting interpersonal harm or interpersonal assistance (30 of each; see Figure 1 for examples). These dynamic visual stimuli have been used in previous investigations of moral reasoning in healthy participants and are reliably judged to be morally bad (Bad) and morally good (Good), respectively.^49, 70^ Briefly, three static images are extracted from videos and presented in succession to create apparent motion (1000, 200 and 1000 ms). Blocks consisted of five scenes of the same type, each followed by a one second fixation cross. There were four runs, each containing three blocks of each type presented in pseudo-random order and interspersed with rest blocks of 14 s. In each block, one randomly selected scene was followed by a question screen (2000 ms). Participants indicated their response ('Yes' or 'No') using one of two buttons. The first two runs were intended to assess implicit moral judgment; therefore, rather than explicitly cuing participants to attend to or evaluate the morally relevant content of the stimuli, the question slide asked whether or not the action occurred indoors ('Was this inside?'). The last two runs assessed explicit moral judgment by asking participants whether the action was morally wrong ('Was it wrong?'). Thus, both tasks required participants to make simple Yes–No choices about morally laden stimuli; however, moral information was only relevant in the later task. Before the start of the run participants were given instructions as to what question they would be asked and reminded of the button–response mappings. The button–response mapping was counterbalanced across participants.

### Scanning parameters

Scans were collected using the Mind Research Network 1.5 Tesla Siemens Magnetom Avanto Mobile unit (Washington, DC, USA) equipped with a 12-element head coil and advanced sequence gradients. Echoplanar imaging images were acquired with a gradient echo pulse sequence (repetition time/echo time=2000 ms/39 ms; flip angle=90 ^o^; in-plane resolution=3.4 × 3.4 mm; slice thickness=5 mm voxels; field of view=240mm × 240 mm; matrix 64 cm × 64 cm). Stimuli were presented via the E-Prime 1.0 software (Psychology Software Tools, Pittsburgh, PA, USA).

### Image processing and analysis

Magnetic resonance imaging images were processed using SPM8 (Wellcome Department of Imaging Neuroscience, London, UK) in MATLAB (MathWorks, Natick, MA, USA). Echoplanar imaging images were realigned, filtered (128-s cutoff), co-registered and normalized to the SPM echoplanar imaging template, and smoothed (8 mm full-width at half-maximum). General linear models were used for statistical analysis. For each model, Block types (Bad and Good) were modeled beginning at the onset of the first picture in the block and lasting until the end of the block (18 s). Motion parameters and run order were included as nuisance regressors. For each Task type (Implicit or Explicit), individual contrast images were generated for the effect of Block (Bad>Good). At the second level, independent sample *t*-tests were used to compare neural differentiation between the high-psychopathy and low-psychopathy groups. All images were thresholded at *P*<0.005 uncorrected with a cluster extent of 10, which has been suggested as an optimal balance between Type I and Type II errors.^71^ In order to investigate the parametric influence of psychopathy scores, the mean percent signal change was extracted from significant clusters from all participants and correlated against their Factor 1, Factor 2 and total PCL-R scores.

To examine functional connectivity, the mean activity was extracted from a 6-mm-radius sphere centered around the right amygdala (*x*=22, *y*=-2, *z*=−16) and right TPJ (*x*=62, *y*=−54, *z*=16), based on coordinates taken from a meta-analysis of functional magnetic resonance imaging studies of morality.^45^ Within each task, the Bad–Good contrast was used as the psychological regressor in separate psychophysiological interaction (PPI) analyses. As before, first-level PPI images were analyzed at the second level with age, IQ, task accuracy and PCL-R scores entered as covariates.

## Results

Descriptive statistics and zero-order correlations among dependent variables are shown in Table 1. IQ was positively correlated with performance on the implicit task (*r*=0.30, Bonferroni-corrected *P*<0.05), but not the explicit task (*P*>0.4). None of the other correlations reached significance after correction for multiple comparisons. Accuracy rates were higher in the explicit task than in the implicit task (*t*(87)=5.42, *P*<0.001, *d*=0.578).

**Figure 2:**
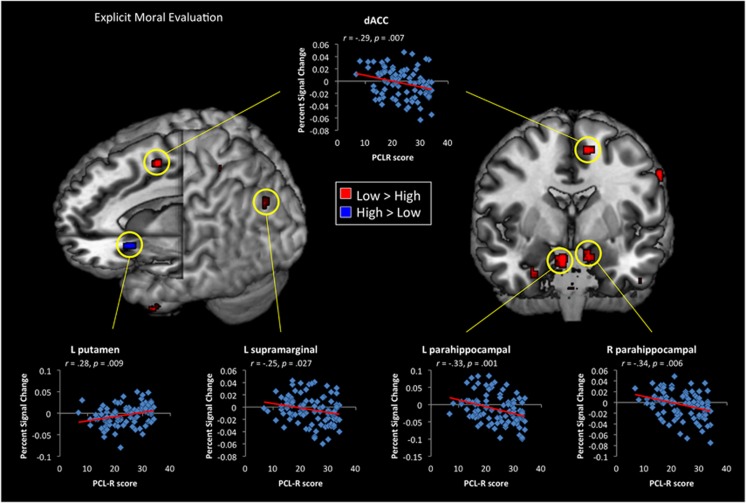
Whole-brain contrasts showing significant (*P*<0.005) differences between High (Psychopathy Checklist Revised (PCL-R)⩾30) and Low (PCL-R⩽20) psychopathy groups during explicit moral evaluations of interpersonal harm versus interpersonal assistance. Scatterplots correlating PCL-R score against percent signal change within specific regions across all participants are shown. dACC, dorsal anterior cingulate cortex; L, left; R, right.

During the implicit task, psychopathy was associated with reduced signal change in the right caudate and dlPFC, but greater signal change in left insula, right temporal pole and subgenual ACC (Figure 1 and Supplementary Table 1). In the explicit task, psychopathy was associated with reduced response in supramarginal gyrus, dorsal ACC (dACC) and large cluster bilateral parahippocampal gyri, extending into left amygdala (Figure 2 and Supplementary Table 2). Conversely, high scores on PCL-R predicted greater activity in the left putamen and right thalamus. Higher scores on PCL-R Factor 1 predicted greater neural response in the dorsal striatum (*x*=−22, *y*=18, *z*=−6) for the interpersonal harm > interpersonal assistance contrast. PCL-R Factor 2 scores uniquely predicted reduced activity in dorsal ACC (*x*=−18, *y*=18, *z*=38).

**Figure 3:**
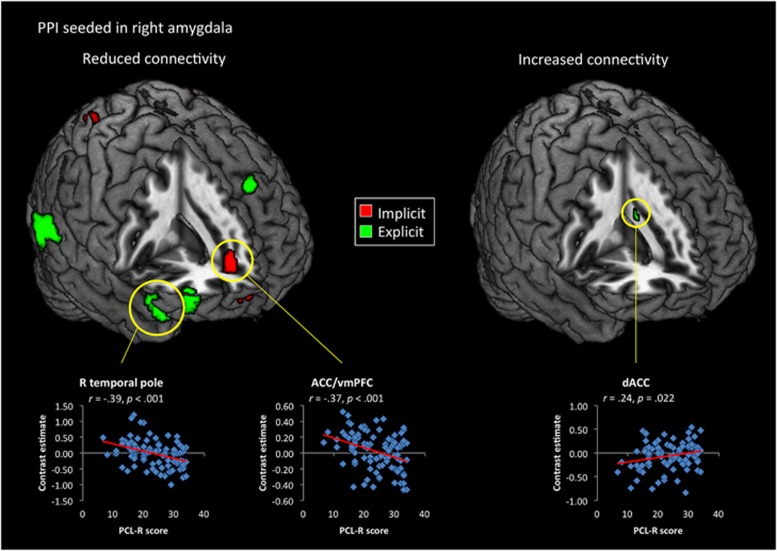
Regions showing significant (*P*<0.005) influences of total Psychopathy Checklist Revised (PCL-R) scores on functional connectivity seeded in the right amygdala. Scatterplots for PCL-R scores and nodes of the salience network are shown below. ACC, anterior cingulate cortex; dACC, dorsal anterior cingulate cortex; PPI, psychophysiological interaction; R, right; vmPFC, ventromedial prefrontal cortex.

The connectivity analysis revealed that psychopathy scores were generally associated with widespread reductions in functional coupling seeded in the right amygdala across both implicit and explicit moral evaluation tasks (Figure 3 and Supplementary Tables 3 and 4). When harm was task-irrelevant, psychopathy was associated with decreased coupling to the midbrain, ACC, left amygdala and striatum, as well as bilateral superior parietal cortex, right superior frontal cortex and posterior cingulate. Similarly, during the explicit task, PCL-R scores predicted reduced functional connectivity to several nodes of the salience network (bilateral striatum, brainstem, right dorsal aINS and right superior temporal pole) and cognitive control network (left superior parietal lobule, right dlPFC and dorsomedial prefrontal cortex). Further, when evaluation of harm was task-relevant, PCL-R scores were positively related to connectivity in left dorsal aINS, inferior parietal lobule and supplementary motor area. No region showed significant positive influences of the PCL-R score on functional connectivity with the right amygdala during the implicit task.

**Figure 4:**
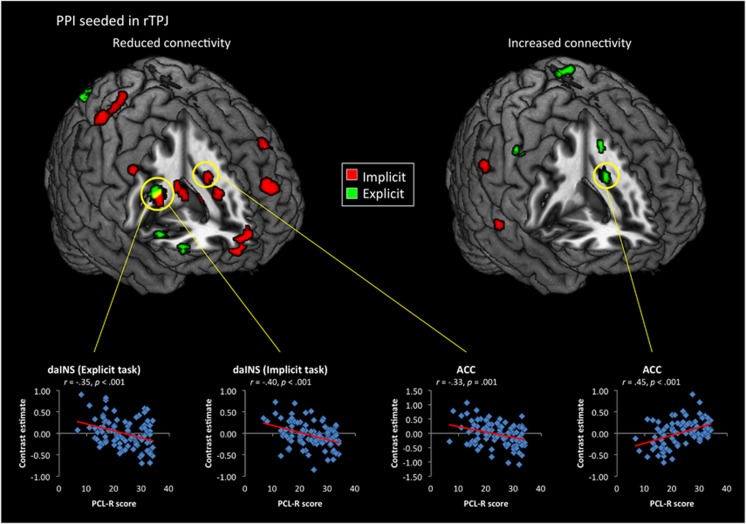
Regions showing significant (*P*<0.005) influences of total Psychopathy Checklist Revised (PCL-R) scores on functional connectivity seeded in right temporoparietal junction (rTPJ). Scatterplots for psychopathy scores and nodes of the salience network are shown below. ACC, anterior cingulate cortex; daINS, dorsal anterior insula; PPI, psychophysiological interaction.

A similar pattern of results obtained for the PPI seeded in rTPJ (Figure 4 and Supplementary Tables 5 and 6). Total PCL-R score was positively related to coupling from rTPJ in both the implicit (right supramarginal) and explicit (dACC and supplementary motor area) tasks. During the implicit task, PCL-R scores predicted significant reductions in connectivity to dACC, right caudate, bilateral inferior parietal lobule and left dorsomedial thalamus in the salience network, as well as several nodes of the cognitive control network, specifically bilateral dlPFC, right anterior thalamus and left frontal cortex. In the explicit task, significant reductions related to the PCL-R score were found in left inferior parietal, putamen, dorsomedial thalamus and right dorsal aINS. Psychopathy was also associated with reduced rTPJ coupling with right dlPFC and superior parietal cortex.

## Discussion

To the best of our knowledge, no study to date has examined the neural response and functional connectivity in both implicit and explicit processing of harm-related moral scenarios in forensic psychopaths. By utilizing ecologically valid depictions of interpersonal harm versus interpersonal assistance, when the harm was and was not task-relevant, the results of this study provide the first direct evidence for the influence of task demands on neural processing of moral information in psychopathy. Although high- and low-psychopathy groups did not differ in their behavioral performance on these tasks, there were several striking differences in the neural networks recruited as well as in functional connectivity, even after controlling for age and IQ. When inmates with high psychopathy scores viewed morally laden scenarios, they showed widespread decreases in functional connectivity seeded in the rTPJ and right amygdala, two important computational nodes previously associated with intention understanding and emotional saliency, which contribute to moral cognition.^49, 72, 73^

As expected, during the implicit task (Figure 1), the low-psychopathy group showed greater activity in dlPFC. This fits well with a previous study with healthy adults who found greater dlPFC activity during implicit compared with explicit moral evaluation.^62^ In healthy individuals, third-person pain is a salient cue that is critical to elicit empathic concern,^74^ and the low-psychopathy group likely spontaneously allocated attention to the cues of harm, even though they were not task-relevant. Indeed, psychopathy scores predicted decreased coupling between the right pSTS/TPJ and aINS, a region that is reliably associated with emotional awareness and empathic processing.^7, 9, 29^ Psychopathy has consistently been associated with an abnormally low aversive response to the distress of others;^3, 75^ thus, greater activity in the dlPFC in the low-psychopathy group during the implicit moral evaluation task may indicate extra mental effort, or compensatory activity, required to inhibit such an automatic response when viewing harmful interactions.^76^

When interpersonal harm was task-relevant, psychopathy, both categorized as a taxon and as a dimensional variable, was associated with increased hemodynamic response in the dorsal striatum (Figure 2). Interestingly, activity in this region was significantly predicted by PCL-R Factor 1 scores. Previous work has linked this region to anticipation of pleasant experiences,^77^ suggesting that individuals with high Factor 1 scores may perceive harmful interactions as more enjoyable. Psychopathy also predicted reduced response in the amygdala and dACC (Figure 2), although dACC activity was significantly correlated with Factor 2, but not Factor 1 (Supplementary Table 2). Unlike previous neuroimaging studies, no whole-brain difference in vmPFC was detected during explicit moral evaluations,^62^ although PCL-R scores did predict reduced functional connectivity between vmPFC and both right amygdala and right pSTS/TPJ (Figures 3 and 4). Further, during the explicit moral reasoning task, higher psychopathy scores predicted decreased response in a left parietal cluster, which extended into left TPJ.

The ACC, because of its unique reciprocal connectivity with subcortical and cortical regions, is an important integrative hub, with dACC in particular exhibiting reliable recruitment during cognitive control, negative affect and nociception.^78^ This overlap across these three domains has been argued to represent a single system whose core function is to determine the optimal response to motivationally relevant situations.^44, 78^ The dACC has also previously been shown to be an important node for orchestrating interactions between widespread cortical networks in service of moral judgment.^52^ Thus, decreased signal in dACC, especially in conjunction with the reduced activity in bilateral parahippocampal gyrus and amygdala, indicate that when explicitly evaluating dynamic visual scenes during moral evaluation, individuals high in psychopathy do not encode interpersonal harm as particularly salient. This fits with previous studies linking psychopathy to reduced amygdala response in social and moral contexts.^7, 8, 36, 79^ However, our results are in contrast with some previous studies suggesting that focusing attention to socioemotional information reduces neuronal differences between controls and psychopaths.^56^ Moreover, given the increased functional connectivity between pSTS/TPJ and ACC, our results may also suggest that other domain-general aspects of information processing are disrupted in psychopathy.

Interestingly, whereas the high-psychopathy group did show greater activity in a cluster in the left superior frontal gyrus, there were only significant group differences in dlPFC during implicit moral reasoning (Supplementary Table 1). Conversely, differential amygdala activation was only observed in the explicit condition (Supplementary Table 2). Thus, the current whole-brain results provide further support for the importance of task demands in determining the amygdala response to moral content. Previous work suggests that explicitly instructing psychopaths to attend to threat stimuli in the context of fear-potentiated startle ameliorates amygdala response by increasing top-down attentional control, primarily via input from lateral prefrontal regions.^56^ However, because group differences in amygdala hemodynamics were only observed in the explicit task, and because these differences occurred without corresponding effects in prefrontal regions, the current results are inconsistent with the hypothesis that psychopathy levels influence amygdala response via increased top-down attentional control,^80^ at least in the current study.

As indicated by the connectivity analysis (Figure 3), individuals with higher psychopathy scores showed reduced neuronal coupling between the right amygdala and vmPFC during implicit moral evaluations. This replicates a similar effect previously observed in incarcerated psychopaths during resting state,^81^ and further supports the argument that disrupted amygdala–prefrontal connections are part of the neurobiological basis of psychopathy.^82^ Moreover, during the explicit task, PCL-R scores predicted decreased connectivity to right temporal pole, inferior frontal gyrus and dorsomedial PFC, but increased connectivity to the dorsal ACC (Figure 4). Given that behavioral performance (that is, subjective evaluations of the scenarios) did not differ between groups, these findings suggest that an increase in psychopathic traits is associated with a shift toward a more focal pattern of network activity in order to leverage the computational capabilities of the ACC. Under both conditions, higher PCL-R scores also predicted reduced neuronal coupling between the amygdala and striatum (Supplementary Table 3 and 4), which is the opposite pattern that has been reported when healthy individuals imagined alleviating the pain of another person.^83^ A similar pattern was observed in the right pSTS/TPJ-seeded connectivity analysis (Figure 4), with PCL-R scores predicting increased connectivity to ACC during explicit moral evaluations, but decreased connectivity with the same region during the implicit task (Figure 4). Psychopathy was additionally associated with increased coupling between pSTS/TPJ and supplementary motor area during the explicit task, but widespread reductions in connectivity to superior parietal cortex, dorsal aINS, ACC, vmPFC and striatum in the implicit task (Supplementary Tables 5 and 6).

Taken together, the findings from the current study provide an initial examination of the influence of task demands on the neural activity in the salience network during moral evaluations in psychopathy. During explicit moral evaluations, psychopathy was marked by reduced activity in several nodes of the salience network, including the amygdala and ACC. Moreover, functional connectivity analyses seeded in the right amygdala and right pSTS/TPJ showed increased coupling to the dorsal ACC. Conversely, when information about interpersonal harm was task-irrelevant, high trait psychopathy predicted reduced activity in dlPFC and decreased connectivity seeded in both the right amygdala and right pSTS/TPJ with ACC, aINS and vmPFC. Overall, functional connectivity analyses can identify patterns of communication between regions that contrast analyses may not detect. Connectivity analyses in our study identified regions whose response covaries with activity in two important computational nodes, amygdala and right pSTS/TPJ, during implicit and explicit conditions, contributing to create a dynamic model of circuits underlying moral evaluation in psychopathy.

## Figures and Tables

**Table 1:** Means and s.d.s for independent variables and task accuracy

	*Full sample*	*Low*	*High*
*n*	94	34	28
Age	31.2 (7.3)	30.1 (7.2)	32.6 (7.4)
IQ	95.2 (12.5)	96.8 (12.6)	94.2 (12.9)
PCL-R	23.7 (7.1)	15.8 (3.3)	31.9 (1.3)
Implicit accuracy	0.88 (0.13)	0.89 (0.12)	0.87 (0.15)
Explicit accuracy	0.96 (0.07)	0.97 (0.05)	0.96 (0.06)

Abbreviation: IQ, intelligence quotient; PCL-R, Psychopathy Checklist Revised.
