# Supplementary material for: Neural networks underlying implicit and explicit moral evaluations in psychopathy
Source: Transl Psychiatry. 2015 Aug 25;5(8):e625–. doi: 10.1038/tp.2015.117 (PMC4564570; doi:10.1038/tp.2015.117)
Supplement: Supplementary Table 1 [file tp2015117x1.doc]

|  |  | MNI coordinates | | |  |  | Correlations | | |
| --- | --- | --- | --- | --- | --- | --- | --- | --- | --- |
| Contrast | Region | x | y | z | Cluster size | T | PCL-R | Factor 1 | Factor 2 |
| Low > High | |  |  |  |  |  |  |  |  |
|  | R Caudate | 4 | 14 | 18 | 26 | 2.96 | -.26* | -.20 | -.25* |
|  | R dlPFC | 34 | 38 | 30 | 38 | 3.45 | -.40** | -.40** | -.47** |
|  | L Precentral | -36 | -6 | 36 | 62 | 3.35 | -.34** | -.29* | -.35** |
|  | L Fusiform | -34 | -70 | -6 | 32 | 3.20 | -.28* | -.28* | -.25* |
| High > Low | |  |  |  |  |  |  |  |  |
|  | R Temporal Pole | 44 | 2 | -32 | 19 | -3.26 | .29* | .22* | .22* |
|  | Subgenual ACC | -2 | 26 | -6 | 41 | -3.30 | .33** | .33** | .21 |
|  | L Insula | -38 | -16 | 10 | 44 | -3.01 | .34** | .24* | .32** |
| Abbreviations: ACC, anterior cingulate cortex; dlPFC, dorsolateral prefrontal cortex; All fMRI clusters significant at p < .005  * FDR-corrected p < .05, ** FDR-corrected p < .01 | | | | | | | | | |

Supplementary Table 1. Group differences during implicit moral evaluation.
