# Supplementary material for: Neural networks underlying implicit and explicit moral evaluations in psychopathy
Source: Transl Psychiatry. 2015 Aug 25;5(8):e625–. doi: 10.1038/tp.2015.117 (PMC4564570; doi:10.1038/tp.2015.117)
Supplement: Supplementary Table 2 [file tp2015117x2.doc]

|  |  | MNI coordinates | | |  |  | Correlations | | |
| --- | --- | --- | --- | --- | --- | --- | --- | --- | --- |
| Contrast | Region | x | y | z | Cluster size | T | PCL-R | Factor 1 | Factor 2 |
| Low > High |  |  |  |  |  |  |  |  |  |
|  | Brainstem | 0 | -28 | -40 | 24 | 3.22 | -.30* | -.36** | -.21 |
|  | R Parahippocampal | 28 | -2 | -32 | 10 | 3.12 | -.33** | -.23 | -.39** |
|  | Cerebellum | -6 | -40 | -30 | 16 | 2.99 | -.27* | -.20 | -.27* |
|  | Pons | 6 | -16 | -24 | 47 | 3.62 | -.36** | -.24* | -.37** |
|  | L Parahippocampal | -12 | -6 | -20 | 48 | 3.38 | -.34** | -.23* | -.31* |
|  | R Lingual | 16 | -96 | -14 | 17 | 3.57 | -.18 | -.12 | -.22* |
|  | L Angular | -34 | -54 | 34 | 18 | 3.22 | -.25* | -.14 | -.27* |
|  | L Postcentral | -60 | -14 | 36 | 27 | 3.18 | -.24* | -.14 | -.26* |
|  | dACC | -18 | 18 | 38 | 37 | 3.13 | -.29* | -.19 | -.27* |
|  | L Superior Parietal | -22 | -42 | 52 | 28 | 3.31 | -.28* | -.27 | -.24* |
|  |  |  |  |  |  |  |  |  |  |
| High > Low |  |  |  |  |  |  |  |  |  |
|  | L Putamen | -22 | 18 | -6 | 33 | -3.84 | .28* | .31* | .26* |
|  | R Thalamus | 16 | -28 | 4 | 11 | -2.96 | .28* | .25* | .22* |
|  | L Superior Frontal | -24 | -12 | 48 | 18 | -2.99 | .34** | .31* | .28* |
| Abbreviations: dACC, dorsal anterior cingulate cortex; All fMRI clusters significant at p < .005  * FDR-corrected p < .05, ** FDR-corrected p < .01 | | | | | | | | | |

Supplementary Table 2. Group differences during explicit moral evaluations.
