# Supplementary material for: Neural networks underlying implicit and explicit moral evaluations in psychopathy
Source: Transl Psychiatry. 2015 Aug 25;5(8):e625–. doi: 10.1038/tp.2015.117 (PMC4564570; doi:10.1038/tp.2015.117)
Supplement: Supplementary Table 3 [file tp2015117x3.doc]

|  | MNI coordinates | | |  |  |
| --- | --- | --- | --- | --- | --- |
| Region | x | y | z | Cluster size | T |
| Caudate tail | -22 | -30 | 26 | 102 | 3.46 |
| L Precentral | -42 | -10 | 42 | 24 | 3.20 |
| Cerebellum | 24 | -30 | -24 | 10 | -2.90 |
| L Amygdala | -22 | 0 | -26 | 11 | -2.96 |
| L Precentral | -26 | -12 | 54 | 16 | -2.99 |
| R Superior Frontal | 20 | 44 | 26 | 14 | -3.00 |
| L Putamen | -18 | 10 | -10 | 32 | -3.00 |
| L Postcentral | -40 | -44 | 62 | 49 | -3.13 |
| L Inferior Temporal | -46 | -8 | -28 | 64 | -3.13 |
| L Inferior Occipital | -22 | -102 | -10 | 16 | -3.17 |
| Cerebellum | -18 | -42 | -32 | 36 | -3.31 |
| OFC | 12 | 56 | -12 | 16 | -3.34 |
| Calcarine | -6 | -102 | -4 | 44 | -3.49 |
| R Superior Parietal | 16 | -64 | 64 | 28 | -3.52 |
| Cerebellum | 14 | -72 | -38 | 24 | -3.64 |
| Midbrain | -2 | -18 | -14 | 175 | -3.87 |
| L Superior Parietal | -20 | -68 | 40 | 63 | -3.88 |
| ACC/vmPFC | 10 | 46 | 8 | 61 | -3.89 |
| PCC | 2 | -40 | 12 | 61 | -4.18 |
| Abbreviations: OFC, orbitofrontal cortex; ACC, anterior cingulate cortex; vmPFC, ventromedial prefrontal cortex; PCC, posterior cingulate cortex  *P* < .005 | | | | | |

**Supplementary Table 3.** Regions showing significant influences of PCL-R score on functional connectivity seeded in right amygdala during the implicit task.
