# Supplementary material for: Neural networks underlying implicit and explicit moral evaluations in psychopathy
Source: Transl Psychiatry. 2015 Aug 25;5(8):e625–. doi: 10.1038/tp.2015.117 (PMC4564570; doi:10.1038/tp.2015.117)
Supplement: Supplementary Table 4 [file tp2015117x4.doc]

|  | MNI coordinates | | |  |  |
| --- | --- | --- | --- | --- | --- |
| Regions | x | y | z | Cluster size | T |
| L Insula | -34 | -18 | 24 | 68 | 3.8073 |
| L Thalamus | -26 | -28 | -4 | 59 | 3.4275 |
| L Inferior Parietal | -34 | -70 | 40 | 70 | 3.4039 |
| SMA | 8 | -16 | 54 | 13 | 3.0279 |
| R Lingual | 28 | -56 | 2 | 19 | 3.0105 |
| L Occipital | -16 | -94 | -20 | 18 | -3.0268 |
| R IFG | 34 | 8 | -14 | 17 | -3.0355 |
| L Middle Occipital | -50 | -80 | 2 | 44 | -3.0668 |
| L Putamen | -18 | 12 | 4 | 18 | -3.1849 |
| L Superior Parietal | -22 | -56 | 68 | 44 | -3.2466 |
| L Middle Occipital | -40 | -68 | 0 | 142 | -3.363 |
| subgenual ACC | 14 | 30 | -10 | 51 | -3.432 |
| R Fusiform | 46 | -28 | -18 | 53 | -3.5718 |
| R Middle Frontal | 46 | 46 | 2 | 46 | -3.5857 |
| L dmPFC | -12 | 36 | 46 | 33 | -3.5875 |
| R Caudate/Putamen | 20 | 18 | -12 | 28 | -3.6277 |
| R Inferior Temporal | 44 | -62 | -6 | 491 | -3.965 |
| L PIC | -34 | -34 | 14 | 58 | -3.9858 |
| R Temporal Pole | 40 | 22 | -22 | 96 | -4.0037 |
| Brainstem | 0 | -30 | -16 | 105 | -4.0642 |
| Abbreviations: SMA, supplementary motor area; IFG, inferior frontal gyrus; ACC, anterior cingulate cortex; dmPFC, dorsomedial prefrontal cortex; PIC, posterior insula cortex  *P* < .005 | | | | | |

Supplementary Table 4. Regions showing significant influences of PCL-R score on functional connectivity seeded in right amygdala during the explicit task.
