# Supplementary material for: Neural networks underlying implicit and explicit moral evaluations in psychopathy
Source: Transl Psychiatry. 2015 Aug 25;5(8):e625–. doi: 10.1038/tp.2015.117 (PMC4564570; doi:10.1038/tp.2015.117)
Supplement: Supplementary Table 5 [file tp2015117x5.doc]

|  | MNI coordinates | | |  |  |
| --- | --- | --- | --- | --- | --- |
| Region | x | y | z | k | T |
| L Postcentral | -46 | -16 | 48 | 28 | 3.58 |
| R Supramarginal | 62 | -36 | 38 | 11 | 2.99 |
| R Superior Temporal | 62 | -24 | 0 | 11 | 2.97 |
| R Superior Temporal | 44 | -32 | 4 | 12 | 2.87 |
| ACC | 6 | 38 | 14 | 11 | -2.85 |
| L Angular | -46 | -64 | 44 | 17 | -2.88 |
| R Postcentral | 28 | -36 | 72 | 24 | -2.91 |
| R Caudate | 12 | 4 | 12 | 26 | -2.94 |
| R Inferior Parietal | 32 | -56 | 50 | 11 | -2.95 |
| L Superior Occipital | -14 | -90 | 28 | 10 | -2.97 |
| R Thalamus | 6 | -14 | 18 | 15 | -2.98 |
| R dlPFC | 36 | 22 | 46 | 25 | -2.99 |
| R Cuneus | 14 | -62 | 20 | 12 | -3.00 |
| Cerebellum | 10 | -72 | -26 | 11 | -3.02 |
| R Precentral | 52 | -2 | 44 | 10 | -3.02 |
| dACC | 14 | 26 | 32 | 36 | -3.05 |
| L Thalamus | -16 | -26 | 16 | 29 | -3.06 |
| aMCC | 6 | 14 | 32 | 18 | -3.07 |
| R Superior Parietal | 38 | -44 | 60 | 26 | -3.09 |
| L Lingual | -10 | -58 | -2 | 54 | -3.10 |
| Cerebellum | -16 | -64 | -32 | 30 | -3.12 |
| R Middle Frontal | 30 | 10 | 52 | 30 | -3.16 |
| L dlPFC | -36 | 32 | 14 | 26 | -3.17 |
| Cerebellum | 24 | -68 | -32 | 48 | -3.19 |
| L Middle Temporal | -50 | -6 | -18 | 29 | -3.20 |
| L Inferior Parietal | -30 | -74 | 48 | 27 | -3.21 |
| L Cuneus | 0 | -70 | 30 | 61 | -3.21 |
| ACC | 8 | 36 | 26 | 33 | -3.25 |
| L Postcentral | -42 | -8 | 34 | 59 | -3.38 |
| L Superior Frontal | -14 | 54 | 26 | 44 | -3.52 |
| R dlPFC | 38 | 46 | 20 | 188 | -3.58 |
| L dlPFC | -34 | 28 | 36 | 68 | -3.59 |
| Cerebellum | 8 | -62 | -14 | 77 | -3.76 |
| vmPFC | 14 | 62 | 2 | 95 | -3.84 |
| R Insula | 36 | 8 | 20 | 103 | -4.84 |
| Abbreviations: ACC, anterior cingulate cortex; MCC, midcingulate cortex; dlPFC, dorsolateral prefrontal cortex; vmPFC, ventromedial prefrontal cortex  *P* < .005 | | | | | |

Supplementary Table 5. Regions showing significant influences of PCL-R score on functional connectivity seeded in rTPJ during the implicit task.
