# Supplementary material for: Neural networks underlying implicit and explicit moral evaluations in psychopathy
Source: Transl Psychiatry. 2015 Aug 25;5(8):e625–. doi: 10.1038/tp.2015.117 (PMC4564570; doi:10.1038/tp.2015.117)
Supplement: Supplementary Table 6 [file tp2015117x6.doc]

|  | MNI coordinates | | |  |  |
| --- | --- | --- | --- | --- | --- |
| Region | x | y | z | k | T |
| dACC | 16 | 22 | 34 | 133 | 4.26 |
| L PIC | -46 | -24 | 22 | 31 | 3.38 |
| aMCC | 14 | -4 | 40 | 34 | 3.23 |
| Precuneus | -14 | -44 | 72 | 23 | 3.21 |
| R Postcentral | 54 | -14 | 54 | 10 | 3.11 |
| SMA | 8 | 18 | 52 | 27 | 3.08 |
| L Postcentral | -56 | -10 | 18 | 10 | 2.92 |
| L IFG | -50 | 36 | 2 | 20 | -2.83 |
| L Lingual | -18 | -60 | -6 | 13 | -2.87 |
| L Inferior Parietal | -40 | -50 | 48 | 12 | -2.89 |
| R IFG | 50 | 42 | 2 | 12 | -2.91 |
| L Superior Occipital | -8 | -94 | 8 | 28 | -2.92 |
| L Fusiform | -32 | -54 | -10 | 13 | -2.98 |
| L Olfactory | -12 | 6 | -16 | 18 | -3.06 |
| PCC | 10 | -42 | 30 | 18 | -3.06 |
| L Inferior Occipital | -34 | -82 | -10 | 15 | -3.08 |
| L Middle Occipital | -34 | -80 | 26 | 11 | -3.15 |
| R Inferior Occipital | 36 | -76 | -8 | 18 | -3.16 |
| L Thalamus | -10 | -14 | 2 | 39 | -3.20 |
| R daINS | 34 | 24 | 8 | 31 | -3.21 |
| R dlPFC | 38 | 40 | 10 | 106 | -3.26 |
| Precuneus | -10 | -72 | 52 | 156 | -3.39 |
| L Hippocampus | -36 | -16 | -20 | 29 | -3.43 |
| L Middle Temporal | -54 | -16 | -10 | 84 | -3.72 |
| MCC | 2 | -20 | 36 | 83 | -3.73 |
| R IFG | 50 | 24 | 10 | 123 | -3.79 |
| R Inferior Temporal | 52 | -54 | -14 | 47 | -3.86 |
| L Putamen | -32 | 6 | -10 | 105 | -3.86 |
| R Superior Parietal | 18 | -74 | 52 | 258 | -3.97 |
| Abbreviations: dACC, dorsal anterior cingulate cortex; aMCC, anterior midcingulate cortex; SMA, supplementary motor area; IFG, inferior frontal gyrus; PCC, posterior cingulate cortex; daINS, dorsal anterior insula cortex; dlPFC, dorsolateral prefrontal cortex  *P* < .005 | | | | | |

Supplementary Table 6. Regions showing significant influences of PCL-R score on functional connectivity seeded in rTPJ during the explicit task.
